# Assessing the Impact of the Different Psychiatric Disorders on the Profiles of Psychiatric Hospitalization: A descriptive study in a Greek Hospital

**DOI:** 10.1192/j.eurpsy.2024.1192

**Published:** 2024-08-27

**Authors:** G. N. Porfyri, P. Papadopoulou, M. V. Karakasi, A. Vlachaki

**Affiliations:** ^1^Psychiatry Department, General Hospital of Papanikolaou, Thessaloniki; ^2^Psychiatry Department, University General Hospital of Alexandroupolis, Alexandroupolis, Greece

## Abstract

**Introduction:**

The prevalence of psychiatric re-admission ranges from 15% to 60%, escalating even more in the first year after admission, affecting the patients’ quality of life. Furthermore, the diagnosis of psychotic or affective disorders represents a risk factor of psychiatric re-admission, highlighting the diagnosis impact to the “profile” of psychiatric hospitalization.

**Objectives:**

To compare the different “Hospitalization Profiles” in association to the patients’ diagnostic categories.

**Methods:**

Overall, 1,633 records of psychiatry inpatients were examined retrospectively throughout the 10-year records of the Psychiatry Department of Papanikolaou General Hospital in northern Greece. The research was conducted between 2013 and August 2023. The sample was divided into subgroups according to gender, diagnoses - according to the International Classification of Diseases (ICD-10)-, and year of hospitalization. A bivariate analysis was performed to examine relationships between the variables: (a.) place of residence; (b.) age; (c.) type of admission; (c.) hospitalization duration; (d.) number of lifetime hospitalizations; (e.) lifetime prosecutor’s orders for coercive examination; (f.) lifetime suicide attempts.

**Results:**

Developmental disorders (F80-89) stood for the youngest average age of hospitalization (26 years) and lowest average hospitalization duration (7 days). Neurodegenerative disorders (F00-09) represented the diagnostic category with the oldest mean age of hospitalization (66 years). Intellectual disorders (F70-79) yielded the longest average hospitalization duration (21 days). Patients with intellectual disorders were found to be facing homelessness at a higher rate (4.76%) than patients of any other diagnostic entity (p=0.096). Psychotic and substance use disorder patients obtained equivalently (p=0.18) the highest rates of coercive hospitalizations (63% and 71%, respectively); compared to other diagnostic categories (p=0.0008). Dual diagnosis and anxiety disorders projected equivalently (p=0.9) the highest rate of premature voluntary discharge (6.9% and 6.4%, respectively). Dual diagnosis, personality disorders, and affective disorders also recorded the highest rates of suicidality (11-15%; with no significant statistical difference among the three diagnostic entities p>0.1) among hospitalized patients of all diagnostic categories (p<0.05).

**Conclusions:**

Interestingly, the study’s results reveal the pathologies of the Greek society, with the most representative example being this of patients suffering from intellectual disorders simultaneously presenting the highest risk of homelessness. Further studies are needed, focusing on the sub-populations of psychiatric patients as well as their status in terms of social security, health care providing, quality of life and life expectancy.

**Disclosure of Interest:**

None Declared

